# Generation of a *de novo* transcriptome from equine lamellar tissue

**DOI:** 10.1186/s12864-015-1948-8

**Published:** 2015-10-03

**Authors:** Heather M. Holl, Shan Gao, Zhangjun Fei, Caroline Andrews, Samantha A. Brooks

**Affiliations:** Department of Animal Sciences, University of Florida, Gainesville, FL 32611 USA; Boyce Thompson Institute for Plant Research, Cornell University, Ithaca, NY 14853 USA; Laboratory of Molecular Immunoregulation, National Cancer Institute, Bethesda, MD 20892 USA

**Keywords:** Equine, transcriptome, laminitis, RNA-seq, assembly

## Abstract

**Background:**

Laminitis, the structural failure of interdigitated tissue that suspends the distal skeleton within the hoof capsule, is a devastating disease that is the second leading cause of both lameness and euthanasia in the horse. Current transcriptomic research focuses on the expression of known genes. However, as this tissue is quite unique and equine gene annotation is largely derived from computational predictions, there are likely yet uncharacterized transcripts that may be involved in the etiology of laminitis. In order to create a novel annotation resource, we performed whole transcriptome sequencing of sagittal lamellar sections from one control and two laminitis affected horses.

**Results:**

Whole transcriptome sequencing of the three samples resulted in 113 million reads. Overall, 88 % of the reads mapped to the equCab2 reference genome, allowing for the identification of 119,430 SNPs. The *de novo* assembly generated around 75,000 transcripts, of which 36,000 corresponded to known annotations. Annotated transcript models are hosted in a public data repository and thus can be easily accessed or loaded into genome browsers. RT-PCR of 12 selected assemblies confirmed structure and expression in lamellar tissue.

**Conclusions:**

Transcriptome sequencing represents a powerful tool to expand on equine annotation and identify novel targets for further laminitis research.

## Background

Laminae are interdigitated dermal and epidermal tissues found in the hooves of livestock that form the attachment to the distal skeleton. Equids have an additional specialization in the form of secondary laminae that project from the primary laminae which further increase the surface area and thus strengthen this connection [1]. The junction between dermal and epidermal laminae must be strong enough to withstand the forces of weight bearing and motion without separation, while providing sufficient flexibility to absorb concussive forces and allow growth. Inflammation of the laminae (laminitis) is a devastating disease that can lead to separation of these tissues and a rotation of the third phalanx (P3) away from the hoof wall.

The etiology of laminitis is poorly understood. Many risk factors have been identified in the horse, including inflammation in other parts of the body, sepsis, metabolic conditions, or mechanical stress [2]. Currently, as there are very few treatments available, prevention through avoiding known risk factors is recommended. In the early stages of laminitis (either pre-clinical symptoms or at the onset of lameness), prolonged cooling of the hooves in ice water has been shown to reduce severity of the disease and prevent separation of the laminae [3]. However, if adequate treatment is not provided promptly, euthanasia is often the result. A study from the United States Department of Agriculture in 1998 estimated the annual cost of lameness at $678 million, with laminitis accounting for 15 % of the reported cases [4]. The American Association of Equine Practitioners has specifically identified laminitis as the disease most frequently reported as needing more research [5].

Several methods have been devised to experimentally induce laminitis, including carbohydrate overload, oligofructose overload, and black walnut extract administration. Although all of these models will result in the disease, key differences in physiological response (as compared to the natural etiology) have been demonstrated [6, 7]. However, as natural cases can be much more difficult to acquire, these models continue to serve an important role in research.

Gene expression has been applied in studies to better understand the disease process. However, much of this research has focused on the expression of few known genes, using qPCR to target specific pathways [8–11]. Only two studies have attempted a transcriptome-wide view of laminitis. The first commercially available whole-transcriptome equine-specific microarray was not published until 2009, therefore early studies attempted two different approaches. The first study chose to use cross-species hybridization with the bovine gene expression chip, identifying 155 out of the 15,000 genes assayed to be significantly up-regulated [12]. They were unable to identify any down-regulated genes, which was likely due to the high false-negative rate associated with imperfect hybridization. A second study instead generated a custom equine-specific array with 3076 targets derived from leukocyte EST libraries [13]. Less than 100 of these genes were found to have significant differential expression.

Both of these projects, and any current work utilizing microarrays, are hindered by insufficient genome annotation in the horse. The only major annotation attempt used an older sequencing technology, generating 35 bp reads from eight diverse tissue types [14]. They identified that 48 % of genes displayed tissue-specific expression patterns, with 7 % of the genes only found in one tissue type. However, this data was not incorporated into automatic annotation pipelines for the popular genome browsers, and lamellar tissue was not included in sequencing. Using this data, the authors also demonstrated there were 428 genes completely lacking in equine annotation, even though many of these genes have data in other species [15].

Whole transcriptome sequencing (RNA-seq) is a promising solution for interrogation of gene structure and expression, especially in a divergent tissue like the hoof. RNA-seq is a hypothesis-free examination of all cDNA in a given sample, allowing for the identification of unique features such as unannotated transcription, splice sites, allele-specific expression, anti-sense expression, and alternative poly-adenylation [16–18]. Additionally, technical variation is reportedly low, with high reproducibility between lanes [19]. Studies have continuously demonstrated high correlation between microarray differential expression studies and RNA-seq strategies, noting the main difference is improved sensitivity for low-abundance transcripts by RNA-seq [20, 21]. However, as RNA-seq is still considerably more expensive and computationally intense than microarrays, much mainstream research still relies on microarrays or qPCR.

The objective of this study was to produce a transcriptome resource for the study of laminitis. Given that recent studies rely heavily on qPCR, the generation of a set of equine, hoof-specific transcripts can greatly benefit in the selection of novel targets for expression studies. Current annotation is largely based on computational predictions and gene models from other species, among which there is not a good physiological model for the laminae. Additionally, while there have been a few equine RNA-seq studies, raw data is often only placed in public databases and not fully processed or curated [14, 22–24]. Thus these valuable datasets are difficult to access and may require intensive bioinformatic analysis before use in subsequent projects, and sadly are often underutilized.

## Results

### Illumina sequencing and assembly

**Fig. 1:**
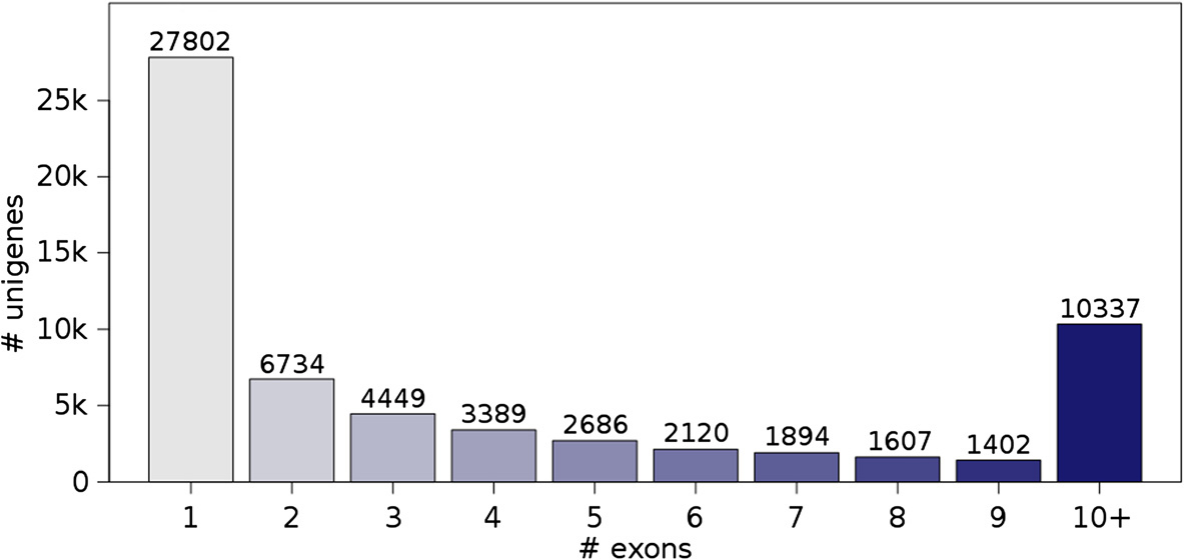
Distribution of exon counts within the unfiltered assembly. Longer models range from 10 to 119 exons

Whole transcriptome sequencing of the three samples in this experiment generated a total of 112,979,003 reads. Sequencing data from all three individuals was pooled for assembly in order to capture genes that may be rare or unique to the laminitic state. After filtering, 87,598,529 high-quality reads remained. The iAssembler pipeline was used to correct for misassemblies due to heterozygosity (either within or between individuals) [25]. A summary of assembly metrics can be found in Table 1 [26]. The number of unigenes (unique transcripts) mapped per locus ranged from 1 to 139, averaging 2.44 isoforms representing 25,580 loci. Many of these unigenes are shorter transcripts covering only a single exon or splice junction, partially due to low-expression transcripts lacking sufficient coverage for assembly (Fig. 1). Considering only the longer 3+ exon transcripts resulted in similar statistics (Table 2).

**Table 1:** D*e novo* assembly statistics

Metrics	Raw Assembly
Total reads (100 bp)	112,979,003
Reads after filtering	86,275,849
Average read length after filtering	88.3 bp
# Unigenes	74,860
N50	2,272
Minimum Length	201
Average Length	1,098
Maximum Length	17,667

**Table 2:** Isoform statistics by locus. Unigenes are clustered together into unique loci based on an overlap of at least 1 bp

Statistics	All Transcripts	Long (3+ Exon) Transcripts
Total Unigenes	55,120	27,884
Unique Loci	23,779	12,905
Min Unigenes per Locus	1	1
Max Unigenes per Locus	125	89
Average Unigenes per Locus	2.32	2.16

Overall, 88 % of raw sequencing reads mapped to the equCab2 reference genome [27]. The GATK recommended pipeline identified a total of 131,034 SNPs [28–30]. We filtered the assembly to remove any alignments matching repeat regions, and then removed SNP calls that fell outside of our transcript models, reducing potential false positive SNPs resulting from incorrectly mapped spliced reads. The 119,430 SNPs that remained (91.1 %) were submitted to dbSNP at NCBI (Table 3).

**Table 3:** Mapping statistics for RNA-seq onto the equCab2 reference genome assembly

Sample	Phenotype	Total Reads	Mapped Reads	% Mapped	SNPs
CU1	control	36,277,643	31,561,549	87 %	60,580
CU18	acute	43,422,463	38,211,767	88 %	72,281
LSU-J	chronic	33,278,897	29,618,218	89 %	58,368

### Annotation with known gene and protein databases

Using blastx, a total of 36,195 unigenes (48 %) matched to proteins in the non-redundant database (significance defined as an E-value less than 1e-5). To simplify the analysis, only the top hit for each unigene was retained. 35 % of the matches were to equine proteins, and of these, 97 % were computationally derived entries (XP_ accession numbers). Additionally, unigenes aligned by BLAT to the equine genome were compared to the NCBI horse RefSeq, NCBI non-horse RefSeq, and Ensembl prediction tracks available from the UCSC Genome Browser. A summary of overlap between the known databases is provided in Table 4.

**Table 4:** Unigenes matching records in selected databases. The repeat-filtered assembly was utilized for EquCab2 alignment-based annotation

Database	Total Records	Unigenes
NCBI NR Protein	37,818,139	36,195 / 74,860^a^ (48 %)
Equine-Specific Repeats	2,905,169	19,740 / 74,860^a^ (26 %)
Non-Horse RefSeq	255,606	24,501 / 55,120^b^ (44 %)
Ensembl Predictions	29,196	15,538 / 55,120^b^ (28 %)
Horse RefSeq	1,169	604 / 55,120^b^ (1 %)
None	n/a	31,091 / 74,860^a^ (42 %)

^a^Unfiltered transcriptome assembly

^b^Repeat-filtered transcriptome assembly

**Fig. 2:**
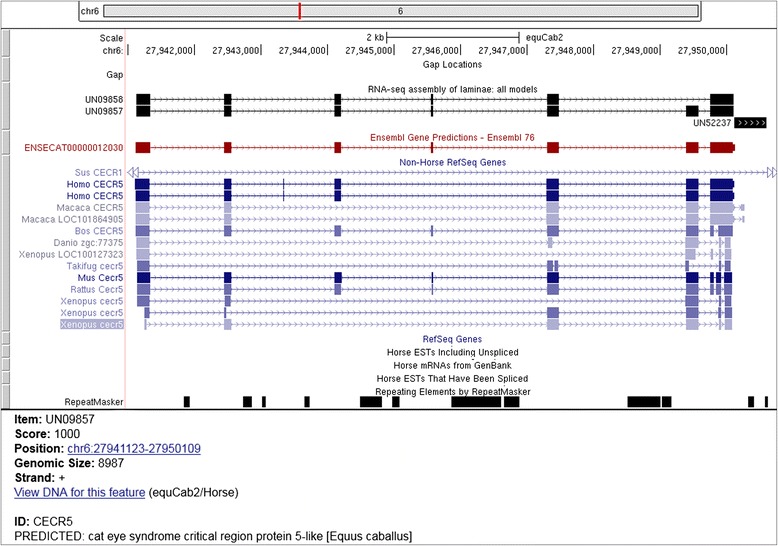
Example custom annotation on UCSC Genome Browser. Clicking on the identifier “UN09857” loads the screen in the lower panel. Custom identifiers provide corresponding gene and protein annotation for each unigene

Gene IDs were assigned to each unigene based on matches to the non-redundant protein database or RefSeq alignments, resulting in annotation of 44,730 transcripts. Unannotated transcripts retained their identifier provided by Trinity. These transcripts likely correspond to novel genes or non-coding RNA and were selected for further examination. This annotated alignment can be loaded into commonly used genome browsers to supplement existing annotation (Fig. 2) [31].

### Amplification and sequencing of cDNA from putative novel transcripts

There were a total of 13,632 unigenes with 3 or more exons that did not match to known RefSeq annotation. Of these, there were 4,718 that did not overlap with other unigenes. A subset of 12 unique transcripts that contained ORFs which spanned over 3 exons were selected for molecular validation (Table 5).

**Table 5:** Putative novel loci validated by RT-PCR

Name	Chr	Start	End	ORF length	Exons	E	BLASTX
UN20159	1	160767946	160771835	85	4	1.2E-40	AAA80518 T-cell receptor alpha chain (IgC TCRA) [Equus caballus]
UN14299	4	24422341	24427035	83	4	none	none
UN30143	5	15325547	15363449	515	16	7.3E-22	XP_003209297 PREDICTED: intraflagellar transport protein 80 homolog [Meleagris gallopavo]
UN27297	5	74952780	74965671	177	6	1.7E-56	XP_001493637 PREDICTED: guanylate-binding protein 5 [Equus caballus]
UN27113	9	81709438	81711119	137	4	2.9E-60	XP_001917082 PREDICTED: lymphocyte antigen 6H-like [Equus caballus]
UN28086	11	880333	894532	240	6	9.9E-31	NP_663348 secreted and transmembrane protein 1A precursor [Mus musculus]
UN62514	11	37045433	37058857	162	5	1.5E-11	ACI67873163 Perlwapin [Salmo salar]
UN21936	12	19828520	19834360	195	5	2.1E-14	NP_001243909 placenta-specific protein 1 precursor [Equus caballus]
UN70945	22	34309639	34312469	141	4	1.7E-54	AES10462 antileukoproteinase-like protein [Mustela putorius furo]
UN26965	24	44802220	44806274	142	5	3.5E-21	XP_002696828 PREDICTED: uncharacterized protein LOC509029 [Bos taurus]
UN26584	28	21567866	21620053	248	7	5.8E-102	XP_003952339 PREDICTED: uncharacterized protein LOC101059192 [Pan troglodytes]
UN50658	X	2231083	2240297	174	7	1.0E-38	XP_003134963 PREDICTED: odorant-binding protein-like [Sus scrofa]

**Fig. 3:**
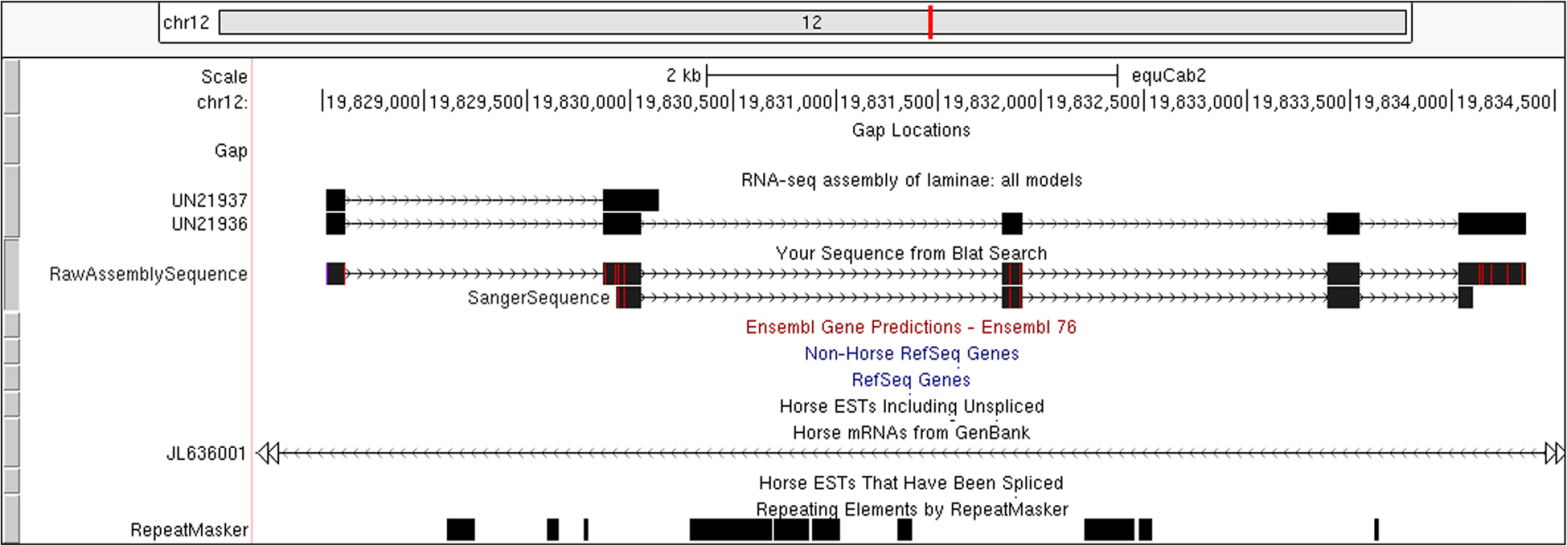
BLAT alignment of sequenced cDNA from UN21936 and assembled transcripts to the reference genome. Screenshot was captured from the UCSC Genome Browser. Dark boxes represent exons while thin lines are introns. The empty RefSeq Genes, Ensembl Gene Predictions, Horse ESTs, and Non-Horse RefSeq tracks indicate that there has never been expression or computational predictions placed here. Although the amplicon shows three mismatches to the reference (shown as vertical red lines on the SangerSequence data), this sequence aligned perfectly to the sequence from the *de novo* assembled transcript (RawAssemblySequence)

**Fig. 4:**
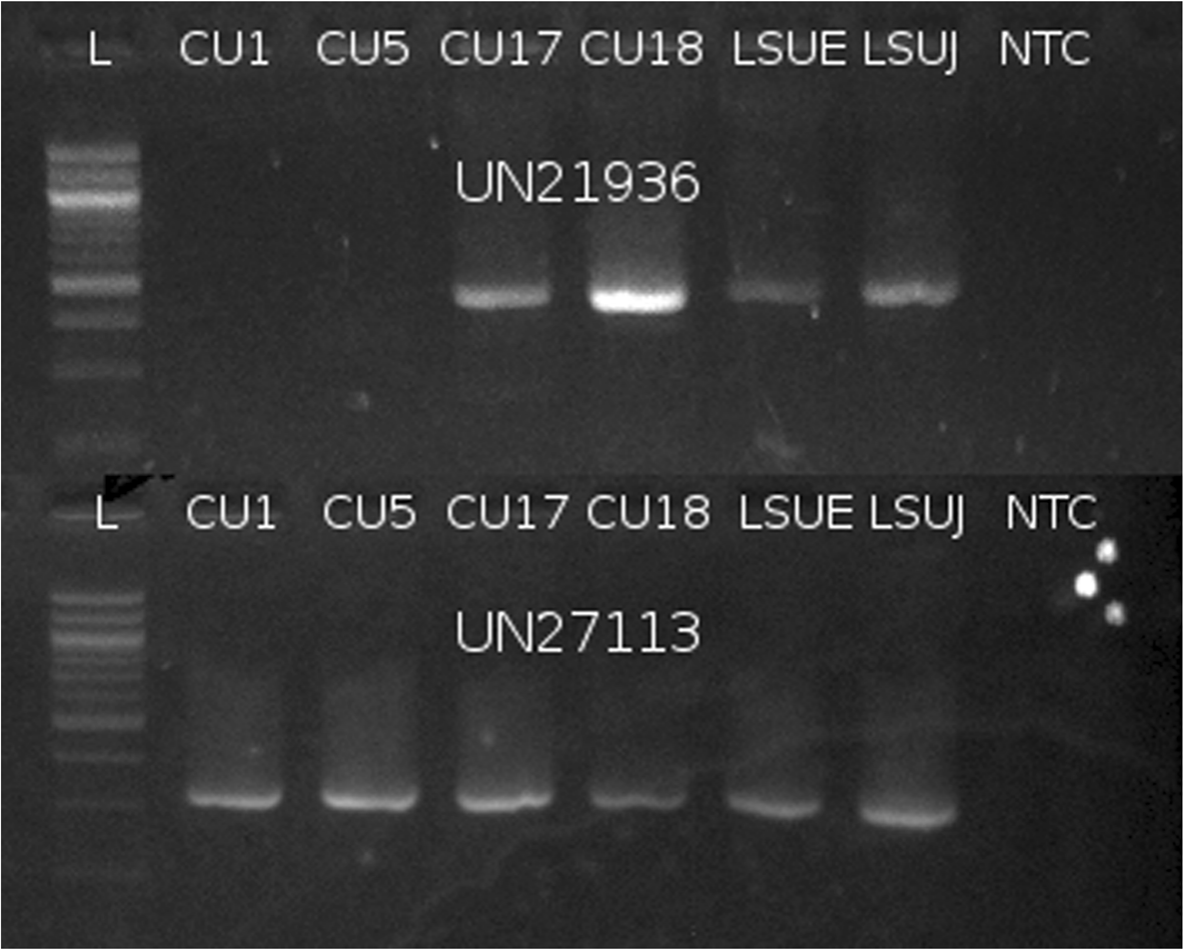
Agarose gel demonstrating the expression of UN21936 and UN27113. Expression of UN21936 appears to be limited to laminitic samples. CU1, CU5 = control; CU17, CU18 = acute laminitis; LSUE, LSUJ = chronic laminitis; NTC = non-template (negative) control

RT-PCR successfully amplified cDNA from all selected transcripts. All products were of the expected length and Sanger-derived sequences matched completely with assembled sequences (example in Fig. 3). As differential expression was not the goal of this study, no quantitative analyses were attempted. However, one selected transcript did display a qualitative trend for disease-specific expression (Fig. 4). The best protein match (placenta-specific protein 1 precursor), located on ECAX, is a computational prediction with support from 1 equine mRNA and 85 % coverage of RNA-seq alignments from one sample in the short-read archive. The only other equine-specific protein match was to a homologous gene, placenta-specific protein 1-like (E = 4e-9), which was mapped approximately 100 kb downstream of the unigene alignment on chromosome 12. However, this record is completely computationally derived, supported only by similarity to two proteins.

## Discussion and conclusion

We utilized RNA-seq to successfully generate a transcriptome assembly of equine lamellar tissue. As the hoof is a specialized tissue, it likely has unique transcripts that previous annotation efforts would have missed. By pooling data from healthy and diseased tissues, we have captured loci that should be valuable to future differential expression studies. Though the varied physiological states could result in differences between each transcriptome, pooling the data prior to assembly ensures sufficient power to assemble lower expressed loci. This data set represents a valuable tool for laminitis research, providing information on both known genes expressed in the hoof, as well as a wealth of previously unannotated transcripts. The transcripts identified in this study can now be utilized with other technologies to search for novel targets with relevance to laminitis.

RNA-seq provides unprecedented power for transcript and isoform discovery. However, relatively little of this information trickles down to human-readable annotation and applied datasets useful to the average molecular biologist. While some resources now exist that attempt to bridge this gap by providing bioinformatics instruction for molecular biologists, this approach is not practical for all researchers [32]. Our newly generated data is available in two ways. Raw reads and identified variants have been deposited in public databases, so that they may be accessed or incorporated into automated pipelines. NCBI has recently begun to advantageously incorporate RNA-seq data from the short-read archive into their RefSeq annotation pipeline, and the inclusion of additional unique tissue types is essential for robust annotation from this automated analysis. However, these updated annotations (especially computational predictions) are not always readily accessible in popular genome browsers. Therefore, we have also provided downloadable Browser Extensible Data (BED) tracks of our assembly. The first file, labeled the “full” assembly, includes models of any number of exons. We have also provided only those models with 3 or more exons (the “larger” assembly) in order to remove partial transcripts likely originating from poorly expressed loci and intronless non-coding RNAs. The BED format is small and much easier to use than the raw sequencing data itself, including only the positions of each feature (not the exact sequence). BED files also are quite easy for individual researchers to load gene model annotation into their browser of choice [33].

Our data also includes potential non-coding RNAs, which are an emerging field of research. As the RefSeq set is specifically designed for protein-coding genes, all other transcript types are not given accession numbers. There are existing databases of non-coding RNAs available for the human and mouse genomes, however for all other species, there are only the few (less than ten) entries manually curated from the literature [34]. Unlike protein-coding genes, there is considerably less sequence conservation between species in non-coding RNAs, necessitating within species identification [35]. Within non-coding RNAs, there are two main classes: small (<200 bp) and long (>200 bp) [36]. While long non-coding RNAs are often picked up in normal RNA-seq experiments (and must be separated from protein-coding mRNAs for analysis), the smaller molecules are often excluded in normal RNA-seq library preparation, and require additional methodologies to sequence.

The function of non-coding RNAs has been the subject of recent controversy. It is debatable whether the observed RNA transcription is biologically relevant, or if transcription may simply be technical noise [37, 38]. Well documented functions for non-coding RNA include regulation of the genome (through chromatin modification, DNA binding, and protein binding) and of cellular differentiation during development [39–41]. One of the most well-known non-coding RNAs is *XIST*, which regulates X chromosome inactivation in females. More recently, several mutations that cause overexpression of a conserved long non-coding RNA proved to be responsible for the bovine polled phenotype [42]. It is thus important to consider all possible RNAs in studies of differential expression, instead of only the protein-coding transcripts.

Utilization of this data in studies of laminitis could identify new targets and pathways to help further our understanding of the etiology. Whereas current veterinary methods generally can only detect laminitis at the onset of lameness, the development of biomarkers could allow for rapid identification (and thus the most effective treatment) of cases before permanent damage occurs. Future understanding of the precise pathways underlying laminitis could lead to vital novel prevention methods and treatments.

## Methods

### Sample collection and transcriptome sequencing

Samples were collected from four horses presented for necropsy for disposal to the Cornell University College of Veterinary Medicine (samples labeled CU). An additional two lamellar samples were provided by a collaborator (labeled LSU). Medical history was collected when available. Full-thickness, mid-sagittal hoof sections were placed on ice for transport to the lab, gross examination, and dissection of lamellar tissue. Samples were placed into RNA later (Life Technologies, Carlsbad, CA, USA) and stored at -80°C until processing.

Phenotype was assessed through medical history, physical exam prior to euthanasia, and gross findings. Control animals were defined by the distal phalanx running parallel to the hoof wall, with no bruising or thickening of the laminae. Acute cases often had some degree of rotation and/or sinking, as well as lamellar hemorrhage, edema, and thickening. Chronic cases were defined by thickened, fibrous lamina; variable resorption and/or remodeling of the distal phalanx, often with rotation and/or sinking; and variably severe chronic hemorrhage. Sample information can be found in Table 6.

**Table 6:** Summary of samples used in this study. Laminitis phenotype was determined through medical history and histological examination. Demographic information was not available for most horses

Sample ID	Phenotype	Age	Sex	Breed	History	Experiment
CU1	control	unknown	unknown	unknown	healthy	RNA-seq, RT-PCR
CU5	control	3 years	gelding	Quarab	healthy	RT-PCR
CU17	acute	13 years	stallion	unknown	enterocolitis	RT-PCR
CU18	acute	unknown	unknown	unknown	enterocolitis	RNA-seq, RT-PCR
LSU-E	chronic	unknown	unknown	unknown	Equine Metabolic Syndrome	RT-PCR
LSU-J	chronic	unknown	unknown	unknown	Equine Metabolic Syndrome	RNA-seq, RT-PCR

RNA was extracted from approximately 60 mg of lamellar tissue using the Qiagen RNeasy kit (Qiagen Inc., Valencia, CA, USA) following manufacturer's protocols for fibrous tissue. 50 μL of RNA was DNase treated using either the Ambion Turbo DNA free kit (Life Technologies, Carlsbad, CA, USA) or Qiagen DNase I kit, followed by Qiagen RNA cleanup kit. Quantification was carried out using a NanoDrop spectrophotometer (NanoDrop Technologies LLC., Wilmington, DE, USA).

Library preparation and sequencing was performed by Cornell University's Life Sciences Core Laboratory Center. A total of 5-10 μg of RNA from each sample was submitted. Single-end libraries were constructed using manufacturer's protocols for poly-T selection and sequenced on an Illumina HiSeq 2000 (Illumina Inc., San Diego, CA, USA). Raw reads were submitted to the European Nucleotide Archive [ENA:PRJEB6100].

### De novo *assembly*

Raw RNA-seq reads were processed in two steps. First, a custom R script (based on the ShortRead package) was used to remove adapter and barcode sequences, as well as to trim low quality (Q < 20) bases from both ends of the reads [43]. Trimmed reads shorter than 25 bp were discarded. Second, reads were aligned to the GenBank virus (version 186) and ribosomal RNA sequence databases with BWA under default parameters [44]. Only unmapped reads were retained for assembly.

The filtered reads from all samples were pooled and *de novo* assembled into contigs using Trinity with “min_kmer_cov” set to 2 [45]. In order to remove some of the redundancy of Trinity-generated contigs, a further assembly step using iAssembler with a minimum of 99 % identity (-p) was performed [25]. Contigs shorter than 200 bp were discarded.

### Unigene annotation

All unique transcripts (unigenes) were compared to the GenBank non-redundant protein database using blastx with an E-value cutoff of 1e-5. Only the protein with the lowest E-value (and thus highest significance) was retained for further analysis.

Unigenes were also aligned to the equCab 2.0 reference genome using BLAT with parameters recommended for same-species mRNA alignments [46]. The pslCDnaFilter tool was used to remove alignments with less than 200 bp, 98 % identity, or 50 % coverage. The resulting PSL file was converted to BED format and compared with Equine-specific repeat annotation using BEDtools intersectBed in order to filter out alignments that contained over 10 % repetitive DNA [47, 48]. Many retroviruses in the genome are expressed, but high homology among these elements often leads to chimeric and spurious assemblies, and thus creates problems for alignment-based analyses. The filtered unigenes were then compared to NCBI Non-Horse RefSeq, Horse RefSeq, and Horse Ensembl annotations using intersectBed at 10 % overlap.

Putative gene names were assigned to unigenes based on high quality matches to NCBI non-redundant databases. Two BED files were produced for use in genome browsers (one containing all transcripts and one with only large transcripts containing 3 or more exons) [31].

### Variant calling

Raw sequencing reads were split by barcode and aligned to the EquCab 2.0 reference genome using BWA under default parameters. SAMtools was used to convert alignments to BAM format and to remove PCR duplicate reads [49]. SNPs were identified with GATK using the recommended pipelines with a Q > 30 cutoff [28–30]. VCFtools was then used to filter out variants with fewer than 10 observations, followed by BEDtools to remove variants that fell outside of regions with corresponding assembly alignments [50]. The final list of variants was pooled and submitted to NCBI dbSNP.

### Analysis of putative novel loci

We screened the transcriptome assembly for novel loci with two steps. First, a second genome alignment was prepared by running RepeatMasker (using RepBase 2013-04-22 libraries) on the unigenes, then BLAT and subsequent filtering was performed as before [51, 52]. Next, the unmasked and masked alignments were compared, and unigenes that passed filtering criteria in both datasets were selected. The unmasked alignments of these unigenes were then compared to RefSeq annotation using BEDtools, and alignments with less than 5 % overlap to known annotation were labeled as putative novel loci. All matches to the unassembled chromosome (chrUn) were discarded. Although valuable novel genes are likely to be found there, the incomplete state of assembly in this region makes downstream alignment based analyses problematic.

Twelve novel genes were selected for RT-PCR validation and proof of concept based on additional criteria. ExPasy “translate” tool was used to identify open reading frames (ORFs) in these unigenes [53]. These were then aligned back to the equCab 2.0 reference genome using BLAT, and only unigenes with ORFs spanning at least three exons on their corresponding transcript annotation were retained, thus identifying larger transcripts with significant exon/intron structure. The ORFs were then compared to the non-redundant protein database using blastp, and targets with little to no experimental data were selected for further validation.

Within each gene, an amplicon of cDNA was targeted using intron spanning primers created with the Primer3 software (Table 7) [54]. Two-step RT-PCR was performed in 15 μL reactions with 1 μg RNA using the SuperScript VILO MasterMix kit (LifeTechnologies, Carlsbad, CA, USA) followed by standard PCR. 1 μL of cDNA was amplified in 10 μL PCR with FastStart Taq DNA polymerase (Roche Applied Science, Branford, CT, USA) and included all reagents per the manufacturers recommended conditions.

**Table 7:** Primers used to confirm expression of unannotated transcripts. All PCRs were performed with an annealing temperature of 62°C and an elongation time of 30s

Name	Forward Seq	Reverse Seq	Size
UN20159	TTCAAGAGCAATGGGATGCT	CGCAGTGTCATGAACAGGTTA	227 bp
UN14299	TTTTCCTCTGAAGCATTTCC	TAGAGCATCGCTTTCCTGGT	284 bp
UN30143	CCCACCCCCAACCTAGATAC	AGGTAAGACAGGCTGGGTCA	499 bp
UN27297	GTCCGAATTCAGCCAATCAT	GAAACGATTTATGGCCTCCA	495 bp
UN27113	TGAAAGGCATCCATCTGGTC	ACCCCGTTACAGAGGTCCTT	329 bp
UN28086	TCCTTGCTAGGATGCTCTGG	GAGCACCAGGATGAAGAGGA	506 bp
UN62514	GGCTCCTCCTCCTTGTGAG	AACAGCAGTTTGGCAGGAGT	437 bp
UN21936	CTATGTTCTGGGCTGTGGTG	TGTAGCCACGTTTGCACTCT	485 bp
UN70945	CCTCATGACCTTCGTGGTTC	ATCTTTTTGAGCTGGCAAGG	409 bp
UN26965	GCACCCTACTCCCACATACG	GCTCACATCCACGTCTGCTA	422 bp
UN26584	GTACATTCCTCCCCTGCAAA	TCGACACCATCCAGTTGAAA	479 bp
UN50658	CTGACCAGGACCCTCAGTCT	TCAGTGACCAGGCCTTCTTC	343 bp

Amplification was verified on 3 % agarose gel, and the resulting PCR products were submitted to the Cornell Core Life Sciences Laboratories Center for sequencing using standard ABI chemistry on a 3730 DNA Analyzer (Applied Biosystems Inc., Foster City, CA, USA). Amplicons were aligned to their corresponding unigenes to confirm identity using Consed [55].

## Availability of supporting data

The data sets supporting the results of this article are available in the NAGRP Host of Supplementary Data to Publications repository, http://www.animalgenome.org/repository/pub/CORNEL2015.0126/.
